# Supplementary material for: A high-resolution mRNA expression time course of embryonic development in zebrafish
Source: eLife. 2017 Nov 16;6:e30860. doi: 10.7554/eLife.30860 (PMC5690287; doi:10.7554/eLife.30860)
Supplement: Supplementary file 6. [file elife-30860-supp6.zip › biolayout-clusters-files/Cluster149-genes.html]

Cluster149


# Cluster149: Genes

| Ensembl ID | Gene Name | Chr | Start | End | Biotype |
| --- | --- | --- | --- | --- | --- |
| ENSDARG00000026842 | acin1b | 7 | 23798266 | 23825852 | protein\_coding |
| ENSDARG00000056621 | ctcf | 18 | 22296926 | 22304306 | protein\_coding |
| ENSDARG00000087094 | foxa | 14 | 33632832 | 33641230 | protein\_coding |
| ENSDARG00000012040 | rnf19a | 19 | 12111226 | 12159073 | protein\_coding |
| ENSDARG00000044485 | sall4 | 23 | 39227796 | 39234669 | protein\_coding |
| ENSDARG00000105508 | si:dkey-23a13.21 | 7 | 5854966 | 5855595 | protein\_coding |
| ENSDARG00000004706 | syf2 | 13 | 45294756 | 45306104 | protein\_coding |
| ENSDARG00000035095 | tdgf1 | 10 | 1927730 | 1934212 | protein\_coding |
| ENSDARG00000016200 | trib3 | 11 | 23820673 | 23825339 | protein\_coding |
